# Neutralizing antibody titers elicited by CoronaVac and BNT162b2 vaccines in health care workers with and without prior SARS-CoV-2 infection

**DOI:** 10.1093/jtm/taac010

**Published:** 2022-02-03

**Authors:** Marcelo J Wolff, Mónica L Acevedo, María Antonieta Núñez, Mónica Lafourcade, Aracelly Gaete-Argel, Ricardo Soto-Rifo, Fernando Valiente-Echeverría

**Affiliations:** Clínica Santa María, Santiago 8389100, Chile; Departamento de Medicina Campo Centro, Facultad de Medicina, Universidad de Chile, Santiago 8389100, Chile; Laboratorio de Virología Molecular y Celular, Programa de Virología, Instituto de Ciencias Biomédicas, Facultad de Medicina, Universidad de Chile, Santiago 8389100, Chile; Clínica Santa María, Santiago 8389100, Chile; Clínica Santa María, Santiago 8389100, Chile; Laboratorio de Virología Molecular y Celular, Programa de Virología, Instituto de Ciencias Biomédicas, Facultad de Medicina, Universidad de Chile, Santiago 8389100, Chile; Laboratorio de Virología Molecular y Celular, Programa de Virología, Instituto de Ciencias Biomédicas, Facultad de Medicina, Universidad de Chile, Santiago 8389100, Chile; Laboratorio de Virología Molecular y Celular, Programa de Virología, Instituto de Ciencias Biomédicas, Facultad de Medicina, Universidad de Chile, Santiago 8389100, Chile

**Keywords:** COVID-19: vaccination, SARS-CoV-2, CoronaVac, BNT162b2, anti-RBD kit

## Abstract

We report neutralizing antibody titers (NAbTs) elicited by CoronaVac and BNT162b2 vaccines in healthcare workers with and without prior SARS-CoV-2 infection using both a pseudotype-based assay and a commercial kit. NAbTs were higher for the mRNA vaccine and increased in all previously infected. Good correlation between both assays was found.

Humoral immune response through neutralizing antibodies (NAbs) elicited by natural infection or vaccination has been shown as critical in the outcome of COVID-19.^1^^,^^2^ NAbs titers vary between different vaccine platforms, and recent findings suggest that individuals with prior SARS-CoV-2 infection receiving viral vector or mRNA vaccines (hybrid immunity) elicit higher titers of NAbs when compared with naïve vaccinees.^3^^,^^4^

The aim of study was to compare NAb response to both vaccines in health care workers with and without prior SARS-CoV-2 infection. Secondarily we proceeded to evaluate comparative performance of a commercial anti-spike RBD kit. All participants signed an informed consent and protocols were approved by the respective Ethics Committee at Clínica Santa María (No. 0802903-21) and Facultad de Medicina, Universidad de Chile (No. 0361-2021). The neutralization assays were performed in 96-well plates using serial dilutions of serum samples and a previously validated assay composed of a bioluminescent HIV-based SARS-CoV-2 Spike Wuhan-Hu-1 (lineage A) pseudotyped virus and HEK293T-ACE2 cells.^5^ Cells were lysed 48 h later, and firefly luciferase activity was measured using the Luciferase Assay Reagent (Promega) in a Glomax 96 Microplate luminometer (Promega). The Elecsys Anti-SARS-CoV-2 S immunoassay is a quantitative ECLIA that detects antibodies against the SARS-CoV-2 spike RBD. Results are automatically reported as the analyte concentration of each sample in U/ml, with <0.80 U/ml interpreted as negative for anti-SARS-CoV-2 S antibodies and ≥0.80 U/ml interpreted as positive for anti-SARS-CoV-2 S antibodies (Roche Diagnostics GmbH. Elecsys Anti-SARS-CoV-2 S assay method sheet. 2021-04; version 1.0). The Elecsys® Anti-SARS-CoV-2 assay uses a modified double-antigen sandwich immunoassay using recombinant nucleocapsid protein (N). It is a total SARS-CoV-2 antibody assay (IgA, IgM and IgG). Results are reported as numeric values in form of a cut-off index (COI) as well as in form of a qualitative results non-reactive (COI < 1.0; negative) and reactive (COI ≥ 1.0; positive) (Roche Diagnostics GmbH. Elecsys Anti-SARS-CoV-2 assay method sheet. 2021-03; version 4.0). Measurement of Anti-SARS-CoV-2 was performed following the manufacturer’s instructions. Statistical analyses were performed using GraphPad Prism software version 9.1.2. Comparisons for neutralizing antibody titers (NAbTs) were carried out using a two-tailed Kruskal–Wallis test after adjustment for the false discovery rate. Correlation analyses were carried out using Spearman’s test. *P* value ≤0.05 was considered as statistically significant.

**Figure 1:**
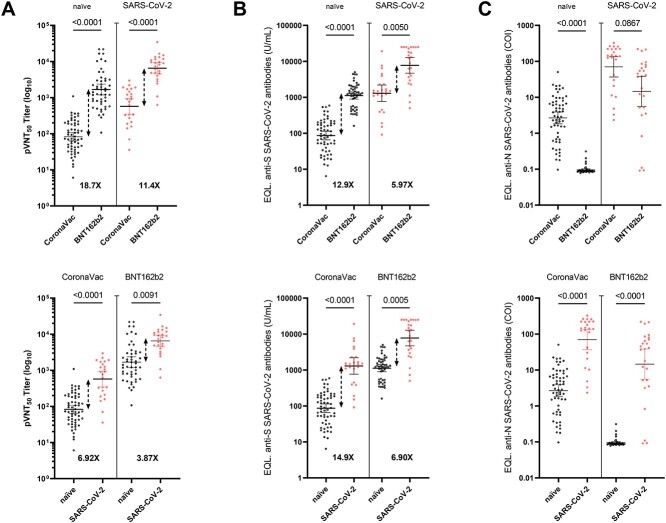
(A) Changes in the 50% pseudovirus neutralization titers (pVNT_50_) in serum samples obtained from the 83 recipients of the CoronaVac vaccine and 75 recipients of the BNT162b2 vaccine, with or without previous infection, against the SARS-CoV-2 Wuhan-Hu-1 (lineage A) variant (upper panel). Data from A showing comparison between vaccine immunity and hybrid immunity (lower panel). (B) Results of serological assay measuring serum reactivity to RBD expressed as U/ml (SARS-CoV-2 positive ≥0.80 U/ml) (Elecsys®, Roche Diagnostic GmbH)) and (C) N protein expressed as COI (SARS-CoV-2 positive cut-off index ≥1.0). Differences in the geometric means titers of neutralization between CoronaVac and BNT162b2 vaccine are shown. Statistical analyses were performed with the two-tailed Kruskal–Wallis test after adjustment for the false discovery rate

To assess the impact of SARS-CoV-2 on vaccine-induced and infection-induced antibodies, we evaluated NAbs titers against the SARS-CoV-2 Wuhan-Hu-1 (lineage A) variant^5^ and determine the performance of a commercial anti-RBD kit (Elecsys® Anti-SARS-CoV-2 S kit, Roche Diagnostics) in a cohort of 158 healthcare workers from Clínica Santa María (Santiago, Chile) being either naïve or reporting a prior SARS-CoV-2 infection diagnosed by RT-PCR and having received a complete vaccination regimen with the inactivated virus vaccine CoronaVac or the mRNA vaccine BNT162b2. Volunteers were classified into the following groups: I) naïve + CoronaVac (*N* = 59); II) SARS-CoV-2 + CoronaVac (*N* = 24); III) naïve + BTN162b2 (*N* = 50) and IV) SARS-CoV-2 + BNT162b2 (*N* = 25). Details regarding cohort demographics, methods and statistical analyses can be found in the Supplementary Tables 1 and 2.

Neutralization assays using the pseudotyped virus revealed NAbs geometric means titers (GMT) of 82.73 (CI: 63.59–107.6), 572.6 (CI: 347.3–944.1), 1681 (CI: 1191–2372), 6521 (CI: 4550–9346) for groups I, II, III and IV, respectively. These results confirm that the mRNA vaccine elicits higher NAbs titers when compared to the inactivated virus vaccine, even in individuals presenting hybrid immunity. Indeed, NAbs titers in volunteers receiving the BNT16b2 vaccine were 18.7-fold or 11.4-fold higher for individuals with vaccine immunity or hybrid immunity, respectively, compared to the respective CoronaVac group (Figure 1A, upper panel). We also observed that volunteers with hybrid immunity presented higher NAbTs compared with those having vaccine immunity (6.92-fold for CoronaVac and 3.87-fold for BTN162b2; Figure 1A, lower panel). Similar results were obtained when anti-spike RBD antibodies were quantified with the Elecsys® Anti-SARS-CoV-2 S kit (Figure 1B). As such, we observed a very strong Spearman’s correlations between the pseudotyped virus neutralization assay and the commercial kit when analysed all the samples (*R* = 0.8505). However, the correlations were moderate to strong (*R* = 0.47, *R* = 0.72, *R* = 0.56, *R* = 0.57) for each group I, II, III and IV, respectively) (Supplementary Figure 1). In addition, we quantified anti-nucleocapsid antibodies using the Elecsys® Anti-SARS-CoV-2 N kit observing that individuals vaccinated with CoronaVac showed some anti-N antibodies as previously reported,^6^ while levels were higher in those previously infected with SARS-CoV-2 (Figure 1C).

NAbs elicited both by natural infection or vaccination have been proposed to serve as a correlate of protection against COVID-19.^1^^,^^2^ In this sense, nation-wide studies showed that CoronaVac, the main component of the national vaccination program in Chile and one of the most widely applied vaccines worldwide,^7^ presented lower effectiveness in preventing symptomatic disease when compared to BNT162b2,^8^^,^^9^ which is in good agreement with differences in NAbs titers reported in our study. Interestingly, recent evidence suggests that prior SARS-CoV-2 infection could act as a booster of the immunogenicity triggered by mRNA and viral vector vaccines.^3^^,^^4^ In agreement with these reports, we showed that NAbs titers elicited by BNT162b2 and CoronaVac are boosted by a prior SARS-CoV-2 infection although at different extents, providing further evidence for hybrid immunity being more potent than vaccine immunity.

Since the characterization of NAb responses under different epidemiological settings is of utmost relevance when planning public health policies related to the management of the COVID-19 pandemic (e.g. the implementation of booster doses in the general population or risk groups), we decided to compare the performance of a commercial kit easily implementable in clinical practice with a validated neutralization assay. Our data revealed a strong correlation, suggesting that some commercial kits detecting total antibodies have the potential to be implemented in general clinics as surrogates of neutralization assays if required.

## Supplementary Material

Wolff_LLJ_Acevedo_et_al_JTM_19Jan_Supplementary_material_taac010
