# Supplementary material for: Neutralizing antibody titers elicited by CoronaVac and BNT162b2 vaccines in health care workers with and without prior SARS-CoV-2 infection
Source: J Travel Med. 2022 Feb 3;29(3):taac010. doi: 10.1093/jtm/taac010 (PMC8903405; doi:10.1093/jtm/taac010)
Supplement: Wolff_LLJ_Acevedo_et_al_JTM_19Jan_Supplementary_material_taac010 [file wolff_llj_acevedo_et_al_jtm_19jan_supplementary_material_taac010.docx]

**Supplementary Appendix**

**Supplementary Figure 1.** Correlation anti-S SARS-CoV-2 serological assay and pseudovirus-neutralizing antibody titer. The *R* and *p* values were determined by two-tailed Spearman’s correlations.

**Supplementary Table 1. Characteristics of study subjects.**

| **Characteristics** | **Total** | | | **CoronaVac** | | | **BNT162b2** | | |
| --- | --- | --- | --- | --- | --- | --- | --- | --- | --- |
| **Group** |  |  |  |  | **I** | **II** |  | **III** | **IV** |
|  |  | **naïve** | **SARS-CoV-2** |  | **naïve** | **SARS-CoV-2** |  | **naïve** | **SARS-CoV-2** |
| **No. of participants** | 158 | 109 | 49 | 83 | 59 | 24 | 75 | 50 | 25 |
| **Age (median, IQR)** | 40 (31-52) | 43 (32-52) | 34 (28.5-51.5) | 40 (31-50) | 40 (33 - 50) | 36.5 (28 - 49.5) | 42 (31-53.25) | 43.5 (31-53.25) | 33 (29 – 54) |
| **Sex** |  |  |  |  |  |  |  |  |  |
| Male (%) | 45 (28) | 35 | 10 | 20 (24) | 14 | 6 | 25 (33) | 21 | 4 |
| Female (%) | 113 (72) | 74 | 39 | 63 (76) | 45 | 18 | 50 (67) | 29 | 21 |

**Supplementary Table 2. Descriptive Statistics**

|  | **ID50 Neutralization** | | | | **Elecys anti-S (U/mL)** | | | | **Elecys anti-N (COI)** | | | |
| --- | --- | --- | --- | --- | --- | --- | --- | --- | --- | --- | --- | --- |
|  | **CoronaVac** | | **BNT162b2** | | **CoronaVac** | | **BNT162b2** | | **CoronaVac** | | **BNT162b2** | |
| **Group** | **I** | **II** | **III** | **IV** | **I** | **II** | **III** | **IV** | **I** | **II** | **III** | **IV** |
|  | **naïve** | **SARS-CoV-2** | **naïve** | **SARS-CoV-2** | **naïve** | **SARS-CoV-2** | **naïve** | **SARS-CoV-2** | **naïve** | **SARS-CoV-2** | **naïve** | **SARS-CoV-2** |
| **n** | 59 | 24 | 50 | 25 | 59 | 24 | 50 | 25 | 59 | 24 | 50 | 25 |
|  |  |  |  |  |  |  |  |  |  |  |  |  |
| **Mean** | 134.2 | 948.6 | 3611 | 8784 | 139.4 | 2624 | 1498 | 12665 | 54.03 | 134.5 | 0.09888 | 6.076 |
| **Std. Deviation** | 161.3 | 806.1 | 5347 | 6872 | 136.3 | 3997 | 1160 | 9704 | 65.68 | 100.2 | 0.03729 | 8.244 |
| **Std. Error of Mean** | 21 | 164.5 | 756.1 | 1374 | 17.75 | 815.9 | 164.1 | 1941 | 13.14 | 20.45 | 0.005273 | 1.073 |
|  |  |  |  |  |  |  |  |  |  |  |  |  |
| **Lower 95% CI** | 92.19 | 608.2 | 2091 | 5947 | 103.9 | 935.8 | 1168 | 8659 | 26.91 | 92.18 | 0.08828 | 3.928 |
| **Upper 95% CI** | 176.3 | 1289 | 5130 | 11621 | 175 | 4311 | 1828 | 16671 | 81.14 | 176.8 | 0.1095 | 8.225 |
|  |  |  |  |  |  |  |  |  |  |  |  |  |
| **Geometric mean** | 82.73 | 572.6 | 1681 | 6521 | 86.55 | 1293 | 1120 | 7729 | 14.51 | 70.15 | 0.09523 | 2.667 |
| **Geometric SD factor** | 2.745 | 3.268 | 3.36 | 2.391 | 2.862 | 3.489 | 2.232 | 3.347 | 10.55 | 4.641 | 1.27 | 4.26 |
|  |  |  |  |  |  |  |  |  |  |  |  |  |
| **Lower 95% CI of geo. mean** | 63.59 | 347.3 | 1191 | 4550 | 65.81 | 762.8 | 891.8 | 4694 | 5.486 | 36.69 | 0.08897 | 1.828 |
| **Upper 95% CI of geo. mean** | 107.6 | 944.1 | 2372 | 9346 | 113.8 | 2191 | 1408 | 12726 | 38.36 | 134.1 | 0.1019 | 3.89 |
|  |  |  |  |  |  |  |  |  |  |  |  |  |
| **25% Percentile** | 42.07 | 225.6 | 742.3 | 4364 | 39.53 | 610.9 | 529 | 3299 | 4.34 | 23.04 | 0.087 | 0.784 |
| **Median** | 73.3 | 838.7 | 1486 | 8225 | 88.67 | 1463 | 1236 | 12339 | 38.17 | 139.6 | 0.089 | 3 |
| **75% Percentile** | 190.9 | 1653 | 3201 | 11674 | 193.1 | 2288 | 1957 | 24803 | 75.13 | 221.4 | 0.09325 | 7.68 |
